# Micro-RNAs, their target proteins, predispositions and the memory of filial imprinting

**DOI:** 10.1038/s41598-018-35097-w

**Published:** 2018-11-28

**Authors:** Giorgi Margvelani, Maia Meparishvili, Tamar Kiguradze, Brian J. McCabe, Revaz Solomonia

**Affiliations:** 1 Institute of Chemical Biology, Ilia State University, Tbilisi, Georgia; 2 I. Beritashvili Centre of Experimental Biomedicine, Tbilisi, Georgia; 3 Department of Zoology, University of Cambridge, Cambridge, UK

## Abstract

Visual imprinting is a learning process whereby young animals come to prefer a visual stimulus after exposure to it (training). The intermediate medial mesopallium (IMM) in the domestic chick forebrain is critical for visual imprinting and contributes to molecular regulation of memory formation. We investigated the role of micro-RNAs (miRNAs) in such regulation. Twenty-four hours after training, miRNA spectra in the left IMM were compared between chicks with high preference scores (strong memory for imprinting stimulus), and chicks with low preference scores (weak memory for imprinting stimulus). Using criteria of significance and expression level, we chose gga-miR-130b-3p for further study and found that down-regulation correlated with learning strength. No effect was detected in posterior nidopallium, a region not involved in imprinting. We studied two targets of gga-miR-130b-3p, cytoplasmic polyadenylation element binding proteins 1 (CPEB-1) and 3 (CPEB-3), in two subcellular fractions (P2 membrane-mitochondrial and cytoplasmic) of IMM and posterior nidopallium. Only in the left IMM was a learning-related effect observed, in membrane CPEB-3. Variances from the regression with preference score and untrained chicks suggest that, in the IMM, gga-miR-130b-3p level reflects a predisposition, i.e. capacity to learn, whereas P2 membrane-mitochondrial CPEB-3 is up-regulated in a learning-specific way.

## Introduction

Successful study of the molecular mechanisms of learning and memory requires that learning-related neural changes and learning-related behaviour are readily measurable. A type of learning offering excellent opportunities for such research is visual imprinting in domestic chicks, whereby a young animal learns the features of an object as a result of being exposed to it^1–3^. Visual imprinting occurs in many species. The intermediate and medial mesopallium (IMM), formerly termed the IMHV^4^, is a chick forebrain region of crucial importance for visual imprinting and the available evidence indicates that it stores information about the imprinting stimulus (reviewed in^2,5^, see also^6,7^). The precise homology of the IMM is unknown. However, the IMM resembles parts of the mammalian association cortex^2^, and homology with neocortical layers 2 and 3 has been proposed based on the distribution of cholecystokinin mRNA expression^8^. The IMM is important in passive avoidance learning in domestic chicks^9,10^. Other parts of the mesopallium are important in several other types of learning. The anterior medial mesopallium has been implicated in auditory imprinting in domestic chicks^11^ and the caudal medial mesopallium in encoding tutor song in songbirds^12,13^.

The strength of learning resulting from imprinting may be measured by a preference score (see Methods), which together with measurements in the IMM permits the identification of neural changes that are specifically related to learning. Criteria used to infer that a change following training is learning-related have been published^14^ (see also Discussion). According to these criteria, learning-related molecular changes have been found to occur in the IMM after imprinting in a progression from transient/labile to trophic modifications, culminating in stable recognition memory^14,15^. These molecular changes include up-regulation of several proteins implicated in neurotransmitter release (clathrin, dynamin-1 and amyloid precursor protein), cellular adhesion (neural cell adhesion proteins and cognin), mitochondrial dynamics (mitofusin-1 and dynamin-related protein-1) and mitochondrial energy metabolism (subunits I and II of cytochrome c oxidase)^16–24^. These data indicate that co-ordinated regulation of gene expression occurs during memory formation.

MicroRNAs (miRNAs) are small non-coding RNAs that are regulators of post-transcriptional gene expression and usually function by base-pairing to the 3′-untranslated regions of mRNA and repressing protein synthesis, blocking translation into protein or destroying the mRNA^25^. miRNAs are important in the regulation of various cellular functions, including synaptic plasticity implicated in learning and memory^26–28^.

We have enquired whether the activity of miRNA and target proteins are modulated in a way specific to learning. We have used miRNA profiling, making no prior assumptions about particular miRNAs. The left IMMs from two groups of chicks were compared: one group (good learners) had developed a strong preference for an imprinting stimulus following training and the other group (poor learners) had developed a weak preference to the same stimulus under identical training conditions. Marked differences between the groups were observed for several miRNAs. Such results do not by themselves demonstrate that the changes are specifically involved in memory and we therefore further investigated the roles of these miRNAs. For one of them, gga-miR-130b-3p, we found down-regulation correlated with learning strength in the left and right IMM after imprinting, but not in a control brain region that is not involved in imprinting. We have also studied changes in the amounts of gga-miR-130b-3p target proteins, chosen from the online database for miRNA target prediction for chicken (http://www.mirdb.org/). One of these targets, cytoplasmic polyadenylation element binding protein 3 (CPEB-3) in a membrane/mitochondrial fraction, showed learning-related up-regulation lateralized to the left IMM.

## Results

Results from both the IMM and the posterior pole of the nidopallium (PPN) are reported in the text. No learning-related effects were found in the PPN; tables summarising results from this region have been placed in Supplementary Tables S2–S4 in Supplementary Material.

### miRNA profiling

The profiling experiment was carried out on the left IMMs of one good learner and one poor learner from each of five batches of chicks. The mean preference score of the good learners was 92.2 ± 2.3 s.e.m, which was significantly higher than the mean preference score of the poor learners (51.8 ± 4.6, matched-pairs t = 27.9, 4 df, P < 0.0001).

For validation experiments, it is recommended to focus on miRNAs showing intensity >500 in at least one of the sample groups (http://www.lcsciences.com/discovery/technical-bulletin-validation-of-mirna-microarray-results-with-real-time-qpcr/). Table 1 shows miRNAs satisfying this criterion. Since the small samples in the profiling experiment allowed only an imprecise comparison of good and poor learners, the miRNA with the most significant result (gga-miR-130b-3p, intensity lower in good learners) was studied using larger sample sizes in the next experiment.

**Table 1:** Results of miRNA profiling experiment, showing log_2_ (mean intensity for good learners/mean intensity for poor learners) and significance of comparison of good learners and poor learners.

miRNA	Log2 (Good learners/Poor Learners)	P value (matched-pairs t test, 4 df)
gga-miR-130b-3p	−0.18	0.05
gga-miR-16-5p	−0.17	0.061
gga-miR-130c-3p	0.47	0.068
gga-miR-19b-3p	0.42	0.074
gga-miR-460b-5p	−0.3	0.075

Normalization using endogenous control genes is currently the most accurate method to correct for potential differences in RNA input or reverse transcriptase efficiency bias (see the above website). We chose two miRNAs as housekeepers (gga-miR-221-3p and gga-miR-99a-5p, Supplementary Table S1, lines 564 and 587 respectively) which, where probability >0.75, had the lowest variance and thus showed the most stable expression.

### Validation for gga-miR-130b-3p

The mean preference score of the 22 trained chicks was 74.3 ± 4.0, significantly exceeding the ‘no preference’ score of 50 (t = 6.0, 21 df, P < 0.0001). Mean approach during training and testing was 75.8 ± 18.5 and 22.45 ± 5.0 metres respectively. Neither approach measure correlated significantly with preference score or with miRNA level and inclusion of these measures in the regression models did not significantly improve the fit; they were therefore dropped. In no analysis was the main effect of the factor Side (left/right) significant. Data analysis for IMM is summarized in Table 2 and for PPN in Supplementary Table S2.

**Table 2:** Standardized relative amount of gga-miR-130b-3p in IMM.

Brain region	IMM, left and right combined		Left IMM		Right IMM	
Housekeeper	miR-221-3p	miR-99a-5p	miR-221-3p	miR-99a-5p	miR-221-3p	miR-99a-5p
Untrained chicks						
Mean	0.97	1.09	1.01	1.08	0.93	1.11
s.e.m.	0.04	0.05	0.05	0.06	0.04	0.08
df	10	10	10	10	10	10
Trained chicks						
Correlation, mi-RNA amount vs preference score	−0.84	−0.84	−0.90	−0.70	−0.69	−0.69
df	10	10	10	10	10	10
P	0.0006*	0.0007*	0.0001*	0.012*	0.013*	0.013*
y-intercept at preference score 100	0.86	1.02	0.82	1.00	0.90	1.03
SE of y-intercept	0.05	0.03	0.04	0.03	0.07	0.04
Comparison, y-intercept at preference score 100 vs mean for untrained						
t	−1.66	−1.31	−2.98	−1.17	−0.40	−0.90
df	18.2	16.6	20.0	15.6	16.7	15.5
P	0.11	0.21	0.01*	0.26	0.69	0.38
y-intercept at preference score 50	1.04	1.15	1.05	1.14	1.03	1.16
SE of y-intercept	0.05	0.03	0.04	0.03	0.07	0.04
Comparison, y-intercept at preference score 50 vs mean for untrained chicks						
t	1.04	0.97	0.70	1.01	1.18	0.59
df	16.2	19.5	18.3	19.7	15.2	19.7
P	0.31	0.34	0.49	0.33	0.26	0.56
Residual regression variance/variance (untrained)	0.21	0.07	0.15	0.17	0.23	0.08
P	0.020*	0.00030*	0.0056*	0.010*	0.030*	0.00035*

Summary of results of regression of gga-miR-130b-3p in IMM on preference score, both housekeepers. Results are also given for untrained chicks. The bottom two rows test whether residual variance from regression with preference score is significantly different from variance of untrained chicks (probabilities are two-tailed). Statistically significant probabilities are starred. In this and the following tables, and in Supplementary Tables S2–S4, numbers of degrees of freedom have been corrected for differences in sample variances.

### gga-miR-221-3p as housekeeper miRNA

**Figure 1:**
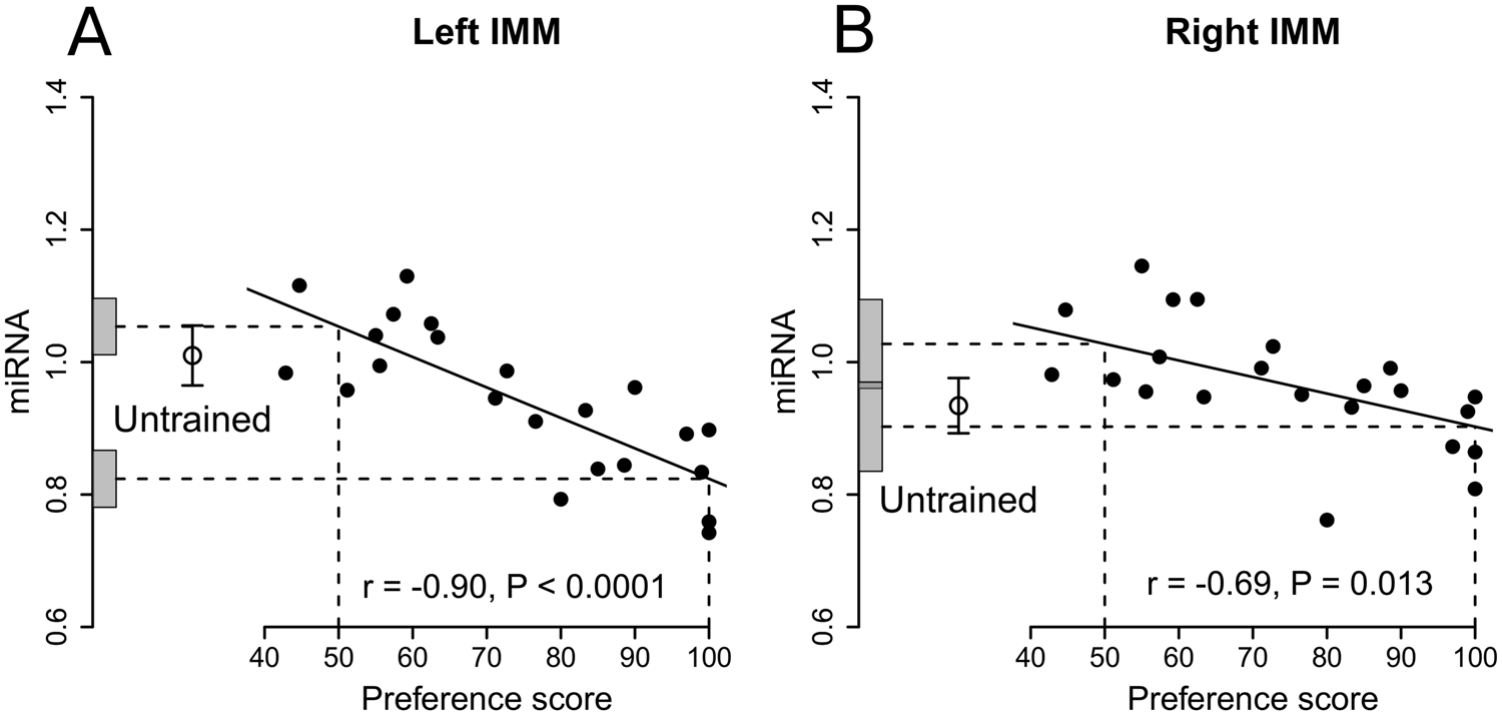
Standardized signal intensity of gga-miR-130b-3p plotted against preference score, gga-miR-221-3p used as housekeeper. Open circles, mean values for untrained chicks ± s.e.m. The horizontal dashed lines denote intercepts on the y-axis corresponding to preference scores 50 (no learning) and 100 (strong learning); ± 1 s.e. of each intercept is shown by the shaded areas along the y-axis. (**A**) Left IMM, significant correlation; the intercept on the y-axis for preference score 50 was not significantly different from the mean for untrained chicks, whereas the intercept for preference score 100 was significantly below the mean untrained value. (**B**) Right IMM; significant correlation. Neither y-intercept was significantly different from the mean value for untrained chicks. Details in Table 2.

When left and right sides were combined, relative amount of gga-miR-130b-3p in the IMM was significantly negatively correlated with preference score (r = −0.84, 10 df, P = 0.0006). There was a significant negative correlation in both the left IMM (r = −0.90, 10 df, P < 0.0001; Fig. 1A) and right IMM (r = −0.69, 10 df, P = 0.013; Fig. 1B), with no significant interaction between preference score and Side. The intercept at maximum preference score was significantly lower (P = 0.007) than the mean for untrained chicks in the left IMM, but not in the right IMM (Table 2). The intercept at preference score 50 did not differ significantly from the mean untrained value in either the left or the right IMM (Fig. 1).

In both left and right IMM, residual variance from the regression with preference score was significantly lower than the variance of untrained chicks (Table 2), whereas the total variance in trained chicks was statistically homogeneous with the untrained values. Taken together with the significant correlations with preference score, these results are consistent with the hypothesis (see Discussion) that the correlations did not arise as a result of training but reflect a predisposition - namely a propensity to learn - that existed in the absence of training.

The correlations between the relative amount of miR-130b-3p and preference score were not significant in the PPN, irrespective of whether the left and right sides were analysed together or separately. The intercepts at maximum preference score and at preference score 50 in the PPN did not differ significantly from the mean amount for untrained chicks. Thus gga-miR-130b-3p level was unequivocally associated with learning strength only in the left IMM.

### gga-miR-99a-5p as housekeeper miRNA

**Figure 2:**
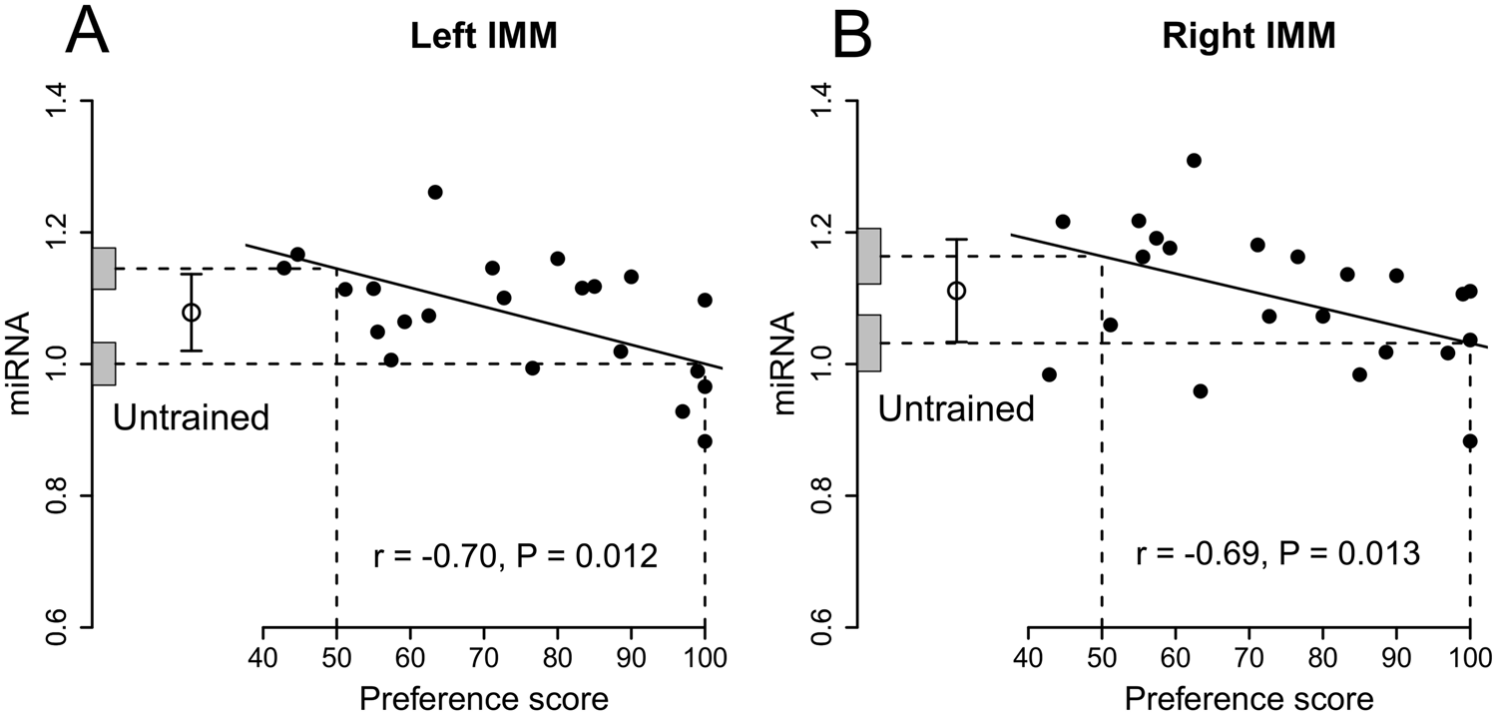
Standardized signal intensity of gga-miR-130b-3p plotted against preference score, gga-miR-99a-5p used as housekeeper. Conventions as in Fig. 1. (**A**) Left IMM, significant correlation. Neither y-intercept was significantly different from the mean value for untrained chicks. (**B**) Right IMM; significant correlation. Neither y-intercept was significantly different from the mean value for untrained chicks. Details in Table 2.

When data from left and right IMM were combined, the correlation between preference score and the relative amount of gga-miR-130b-3p in the IMM was significantly negative (r = −0.84, 10 df, P = 0.0007). Separate analysis by side showed that the negative correlation in the left IMM was also significant (r = −0.70, 10 df, P = 0.012; Fig. 2A) and in the right IMM it was almost identical (r = −0.69, 10 df, P = 0.013, Fig. 2B). There was no significant interaction between preference score and Side. The intercepts at maximum preference score and preference score 50 were not significantly different from the means of untrained chicks on either side of the IMM.

As with the alternative housekeeper, residual variance from the regression with preference score was significantly lower than the variance for untrained chicks in the IMM, whether data from both sides were pooled or analysed separately; in addition, the total variance in trained chicks was statistically homogeneous with the variance of the untrained chicks. These results, with those obtained with the other housekeeper, raise the possibility (see Discussion) that the correlation of miRNA expression with preference score is attributable to a predisposition.

No correlation or difference between intercepts and mean for untrained chicks was significant for the PNN, either for the two sides combined or when they were analysed separately.

### CPEB-1 and CPEB-3

**Figure 3:**
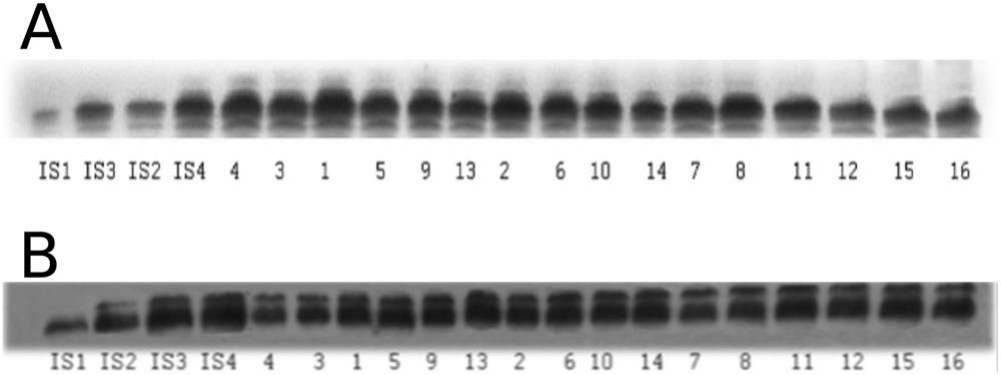
(**A**,**B**) Each comprise sample western blots from four chicks (good learner, intermediate learner, poor learner, untrained control) run simultaneously in a single experimental replication. Each chick contributed four samples (left and right IMM, left and right PPN). (**A**) M-CPEB-1; (**B**) M-CPEB-3. Experimental conditions for both A and B: 1 Good learner, left IMM; 2 Good learner, right IMM; 3 Good learner, left PPN; 4 Good learner, right PPN; 5 Intermediate learner, left IMM; 6 Intermediate learner, right IMM; 7. Intermediate learner, left PPN; 8 Intermediate learner, right PPN; 9 Poor learner, left IMM; 10 Poor learner, right IMM; 11 Poor learner, left PPN; 12 Poor learner, right PPN. 13 Untrained, left IMM; 14 Untrained, right IMM; 15 Untrained, left PPN; 16 Untrained, right PPN. In both A and B IS1 to IS4 corresponds to 15, 30, 45 and 60 µg protein standards from the IMM of untrained chicks (protein amount increases from left to right); these standards were used to calibrate (**A**,**B**) respectively. Note that the effects demonstrated in Fig. 4 are based on statistical analysis of all data and therefore the blots shown here are not necessarily representative of their respective collective results.

CPEB-1 and CPEB-3 were measured in two subcellular fractions of the IMM and PNN: P2 mitochondrial-membrane fraction (M-CPEB-1 and M-CPEB-3) and cytoplasmic fraction (C-CPEB-1 and C-CPEB-3). Antibodies against CPEB-1 reacted with a protein of molecular weight 62 kDa whilst antibodies against CPEB-3 reacted with a protein of molecular weight 75 kDa, corresponding to chicken CPEB-1 and CPEB-3 respectively (Fig. 3).

The mean preference score of the 21 trained chicks was 77.7 ± 3.5 and was significantly higher than the “no preference” score of 50 (P < 0.0001). Mean approach during training and testing were 64.6 ± 12 metres and 17.5 ± 3.7 metres respectively. Neither approach measure correlated significantly with preference score or with any CPEB level and their inclusion in the regression models did not significantly improve the fit; these terms were therefore dropped.

### M-CPEB-1 and C-CPEB-1 (see Table 3 for IMM, Supplementary Table S3 for PPN)

For both subcellular forms of the protein, no correlation coefficient or term in the regression model was significant, either for the whole IMM or PNN or when the data were broken down by side.

### M-CPEB-3 (See Table 4 for IMM, Supplementary Table S4 for PPN)

**Figure 4:**
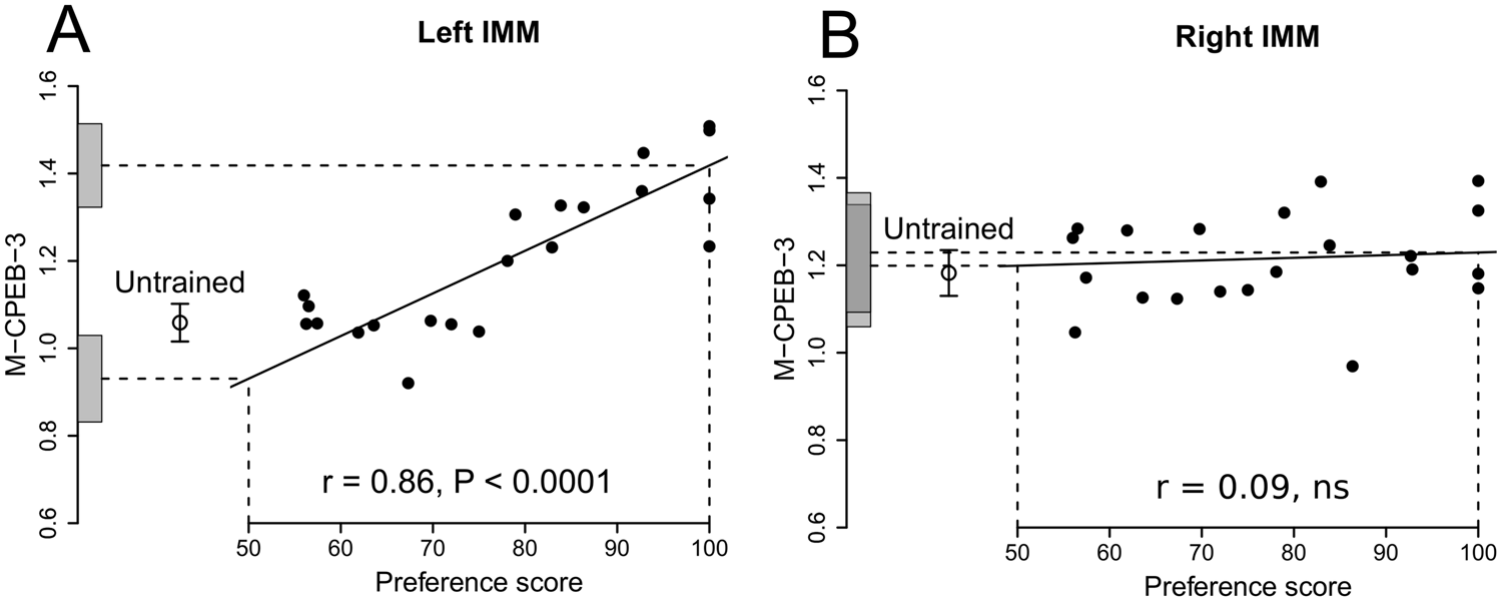
Standardized relative amount of M-CPEB-3 plotted against preference score. Conventions as in Fig. 1. (**A**) Left IMM; correlation significant; the intercept on the y-axis for preference score 50 was not significantly different from the mean for untrained chicks, whereas the intercept for preference score 100 was significantly greater than the mean untrained value. (**B**) Right IMM; there was no significant correlation and neither y-intercept differed significantly from the mean untrained value. Details in Table 4.

When data from the left and right sides of the IMM were combined, the relative amount of M-CPEB-3 was significantly correlated with preference score (r = 0.72, 12 df, P = 0.0034). We further analysed data separately in each hemisphere. A significant positive correlation between amount of M-CPEB-3 and preference score was found only in the left IMM (r = 0.86, 12 df, P < 0.0001; Fig. 4A) and not in the right IMM (Fig. 4B). There was a significant interaction between preference score and Side (F_1,19_ = 9.65, P = 0.006). Thus, the main contributor to the significant correlation in the IMM was the left, rather than the right IMM.

The intercept at maximum preference score was significantly more than the mean for untrained chicks in the left IMM (P = 0.0034), but not in the right IMM (Table 4). The intercept at preference score 50 did not differ significantly from the mean untrained value in either the left or the right IMM (Fig. 4).

In the left IMM, residual variance from the regression with preference score was not significantly different from the variance of untrained chicks. Taken together with the significant correlation between preference score and M-CPEB-3 in the left IMM and the values of the intercepts at preference scores 50 and 100, these results suggest that the correlation in the left IMM arose as a result of training and as a function of the strength of learning (see Discussion).

Since preference score was negatively correlated with miR-130b-3p miRNA expression and positively correlated with amount of target M-CPEB-3 protein in the left IMM, it was of interest to enquire whether the two biochemical measurements were negatively correlated with each other in this region. Such an analysis is not straightforward, since the measurements were made in different groups of chicks. Nevertheless, when each group of chicks was sorted according to their preference scores, the Spearman rank correlation coefficient between miR-130b-3p miRNA expression and amount of M-CPEB-3 was significant and negative (Spearman r = −0.76, n = 21, P < 0.001) with gga-miR-221-3p as housekeeper. With the same housekeeper, a similar result was found in the right IMM, (Spearman r = −0.48, n = 21, P < 0.05). The corresponding correlations were negative but not significant with gga-miR-99a-5p as housekeeper. Correlations across different experiments are potentially unreliable because of high variability between experiments and possible confounding from unknown sources. Therefore, this type of analysis was used only to confirm expected associations between miRNA and target protein indicated by their respective significant correlations with preference score.

In the PPN the correlations between the relative amount of M-CPEB-3 and preference score were not significant, irrespective of whether the left and right sides were combined or analysed separately. The intercepts at maximum preference score and at preference score 50 in the PPN did not differ significantly from the mean amount for untrained chicks. The results thus indicate that the level of M-CPEB-3 varies with learning strength only in the left IMM.

### C-CPEB-3 (See Table 4 for IMM, Supplementary Table S4 for PPN)

For the left and right IMM combined, protein amount was not significantly correlated with preference score. Analysing data separately by side showed no significant correlation in the left IMM, whilst in the right IMM the correlation was marginally significant (r = 0.53, 12 df, P = 0.049). In neither hemisphere did the intercepts at maximum preference score or preference score 50 differ significantly from the mean amount for untrained chicks. No correlation coefficient, or term in the regression model, was significant, either for the whole PNN or when the data were broken down by side. Although residual variance from the regression was significantly greater than variance in untrained chicks for the left and right IMM combined, this was not the case when the data from the two sides were analysed separately (Table 4). No such effect was found in the PPN (Supplementary Table S4).

### Variability in trained chicks

If a measurement is affected by learning, its variability might be predicted to increase with (i) strength of learning, and (ii) duration of training. In order to test prediction (i), we ran correlations between preference score and the squares of residuals from regressions as an index of variation of residuals. No significant correlation was found, for miRNA or either CPEB, in any of the four brain regions. To test prediction (ii), we compared the variance in untrained chicks with the residual variance from the corresponding regression, i.e. in chicks trained for 1 h. When hemispheres were analysed separately, (cf. Tables 2–4 and Supplementary Tables S2–S4), the only significant changes in residual regression variance were reductions, in the case of gga-miR-130b-3p: no significant increases in residual regression variance were found in either the left or the right side. When data from the left and right hemispheres were combined, regression residual variance was significantly greater than variance in untrained chicks for C-CPEB-3 in the IMM (Table 4) and for gga-miR-130b-3p in the PPN (Supplementary Table S2). However, in neither of these two cases was there a significant correlation with preference score and therefore there is no reason to suppose that either is associated with processes related to learning.

**Table 3:** Standardized relative amount of CPEB-1 in IMM.

Brain Region	IMM, left and right combined		Left IMM		Right IMM	
Protein	M-CPEB-1	C-CPEB-1	M-CPEB-1	C-CPEB-1	M-CPEB-1	C-CPEB-1
Untrained chicks						
Mean	1.01	1.12	1.08	1.17	0.93	1.07
s.e.m.	0.07	0.10	0.10	0.16	0.08	0.10
df	7	7	7	7	7	7
Trained chicks						
Correlation, protein amount vs preference score	0.20	0.10	0.42	0.41	−0.03	−0.24
df	12	12	12	12	12	12
P	0.49	0.74	0.14	0.14	0.93	0.42
y-intercept at preference score 100	1.18	1.20	1.27	1.41	1.09	1.01
SE y-intercept	0.08	0.12	0.08	0.15	0.10	0.15
Comparison, y-intercept at preference score 100 vs mean for untrained chicks						
t	1.56	0.50	1.45	1.08	1.22	−0.33
df	18.43	18.89	15.08	17.54	18.96	18.32
P	0.14	0.62	0.17	0.29	0.24	0.74
y-intercept at preference score 50	1.07	1.13	1.04	1.04	1.10	1.25
SE y-intercept	0.09	0.14	0.09	0.17	0.12	0.18
Comparison, y-intercept at preference score 50 vs mean for untrained chicks						
t	0.58	0.06	−0.27	−0.56	1.23	0.88
df	18.35	18.04	18.98	18.76	17.81	16.47
P	0.57	0.95	0.79	0.59	0.24	0.39
Residual regression variance/variance (untrained)	0.89	0.94	0.46	0.49	1.26	2.23
P	0.82	0.89	0.22	0.26	0.79	0.29

Summary of results of regression of CPEB-1 in IMM on preference score. Conventions as for Table 2.

**Table 4:** Standardized relative amount of CPEB-3 in IMM.

Brain Region	IMM, left and right combined		Left IMM		Right IMM	
Protein	M-CPEB-3	C-CPEB-3	M-CPEB-3	C-CPEB-3	M-CPEB-3	C-CPEB-3
Untrained chicks						
Mean	1.12	1.20	1.06	1.16	1.18	1.23
s.e.m.	0.04	0.03	0.04	0.06	0.05	0.05
df	7	7	7	7	7	7
Trained chicks						
Correlation, protein amount vs preference score	0.72	0.30	0.86	−0.01	0.09	0.53
df	12	12	12	12	12	12
P	0.0034*	0.30	<0.0001*	0.96	0.75	0.049*
y-intercept at preference score 100	1.32	1.29	1.42	1.18	1.23	1.38
SE y-intercept	0.11	0.08	0.10	0.12	0.14	0.09
Comparison, y-intercept at preference score 100 vs mean for untrained chicks						
t	1.69	1.05	3.43	0.13	0.32	1.48
df	15.5	14.5	16.2	17.3	15.2	16.7
P	0.11	0.31	0.0034*	0.90	0.75	0.16
y-intercept at preference score 50	1.07	1.13	0.93	1.19	1.20	1.09
SE y-intercept	0.11	0.10	0.10	0.14	0.14	0.10
Comparison, y-intercept at preference score 50 vs mean for untrained chicks						
t	−0.45	−0.67	−1.19	0.19	0.11	−1.26
df	15.0	13.8	15.5	15.6	14.8	15.5
P	0.66	0.51	0.25	0.85	0.91	0.23
Residual regression variance/variance (untrained)	0.52	6.89	0.78	3.07	0.66	1.91
P	0.31	0.017*	0.67	0.14	0.50	0.40

Summary of results of regression of CPEB-3 in IMM on preference score. Conventions as for Table 2.

## Discussion

Expression of gga-miR-130b-3p, the miRNA shown by profiling to discriminate most significantly between good and poor learners, and employing two different miRNAs as endogenous internal housekeepers, was significantly negatively correlated with preference score in both sides of the IMM. Furthermore, the level of expression corresponding to preference score 50 (no learning) was not significantly different from the mean value for untrained chicks: expression of gga-miR-130b-3p only decreased when there was evidence of learning. With gga-miR-221-3p as housekeeper, the intercept at maximum preference score 100 (strong learning) was significantly lower than the mean for untrained chicks in the left IMM: that is, expression was decreased when learning strength was high. On the right side of the IMM, there was a non-significant but suggestive trend reminiscent of previous results, where the correlation was predominant in the left IMM with a similar but weaker effect in the right IMM^15^. Results using the alternative housekeeper (gga-miR-99a-5p) follow a similar pattern, although miRNA expression at the intercept for preference score 100, though lower than the mean untrained value, was not significantly so. For the PPN, no significant correlations were found, either in the left or right PNN or the two sides combined.

Membrane CPEB-3 was significantly correlated with preference score in the left, but not the right IMM. The results raise the question of whether correlation with preference score is a *result* of training and a function of learning strength, or a *predisposition* to learn better, irrespective of training. Our criteria for distinguishing between these two possibilities are based on a pattern of observations that would be expected if the first of these inferences is true, namely that a response is a result of training specifically related to learning. This expected pattern is as follows:-

(i) A significant correlation between the response and preference score.
(ii) No significant difference between the level of response in untrained chicks and the level in trained chicks showing no learning (estimated by the y-intercept at preference score 50). That is, a response unaffected by side-effects of training such as handling, exposure to the training stimulus, locomotor activity, stress, etc.
(iii) A significant difference between response at the maximum preference score measured in trained chicks (typically 100) and the mean value for untrained chicks. That is, increased response at a preference score indicating strong learning.
(iv) No significant reduction in regression residual variance compared with the variance of untrained chicks. If learning were to affect the response in any way, one would expect either no significant change in residual variance (added variance of learning effect too small to be detected), or increased residual variance. Crucially, an effect of learning would not be expected to reduce the residual variance in a linear regression model.
(v) If the effect of learning is pronounced, one would expect an increase in the total variance of trained chicks relative to untrained chicks: the responses in poor learners would be anchored to values characteristic of untrained chicks but responses in good learners would be changed in a learning-related manner. This effect has been observed in some experiments^29,30^ but would not necessarily be detected every time learning changes a response. This is because the effect may be small relative to variation from other sources; an increase in total variance would be expected, but the increase would not necessarily be statistically significant for a given sample size.

This pattern of observations describes the results found for M-CPEB-3 in the left IMM, with a similar, weaker trend in the right IMM. We conclude that levels of M-CPEB-3 change in a learning-related way as a result of training, and therefore that M-CPEB-3 is intimately involved in memory formation.

**Figure 5:**
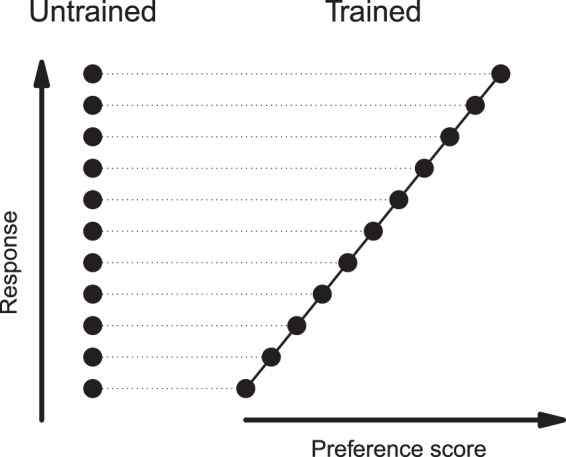
Hypothetical, idealized prediction when a measured quantity (labelled ‘Response’ on the y-axis) determines a predisposition to learn. In the untrained condition, there is a range of values for Response, each point representing the mean for a single animal. Training has no effect on Response. However, because of the predisposition, animals with higher levels of Response show more learning, leading in this extreme, idealized example to a perfect correlation with preference score. The correlation has arisen simply because the original levels of Response were sorted according to preference score/learning strength. All of the variance in the untrained condition is now accounted for by the regression with preference score and the residual variance about the regression line is zero. A significant reduction in residual variance relative to untrained variance thus constitutes evidence of a predisposition.

The expression of gga-miR-130b-3p, particularly with gga-miR-221-3p as housekeeper, follows a pattern suggesting that expression of this miRNA may reflect a predisposition to learn. Figure 5 illustrates an idealised example of this possibility, for a hypothetical sample of measurements, each from a different animal. The animals are trained, but learning has no effect on the measurements. However, the quantity being measured determines a predisposition to learn, in this idealised case resulting in a perfect correlation between response and preference score after training. The correlation arises simply because the measurements have been sorted during training according to the strength of learning. All of the variance before training is now explained by the linear regression with preference score and the residual variance about the regression line has shrunk to zero. In this example, learning has no effect on the measurements and total variance is unchanged. Significant correlation with preference score and significant reduction in residual regression variance are thus consistent with the possibility that expression of gga-miR-130b-3p in the left IMM reflects a predisposition to learn well. The results do not *prove* that a predisposition was present, because additional processes, for which there is not yet evidence, could be operating. Nevertheless, the hypothesis is parsimonious, and identifies residual variance as a source of potentially important information. Furthermore, if correct, it is an example of an miRNA being associated with a predisposition whilst one of its target proteins in the mitochondrial-membrane fraction increases in a learning-related manner as a result of training.

Where there were significant correlations with preference score (Figs 2–4, Tables 2 and 4), it was of interest to investigate variability about the regression line, to determine whether the residual variance from the regression was affected by learning strength or by training time. Residual variance was not significantly correlated with preference score for any quantity measured; there is therefore no evidence for an effect of learning strength. Residual variance for gga-miR-130b-3p was significantly reduced in the IMM of trained chicks (Table 2) and the interpretation of this result in terms of a predisposition is discussed above. Residual variance for M-CPEP-3 was not significantly changed by training, i.e. it was unaffected by training time. It is possible that any effect on residual variance was too small to be detected. Alternatively, predispositions, according to the process described above and in Fig. 5, may have reduced residual regression variance, counteracting the learning-related effect on M-CPEB-3 amount indicated in Fig. 4.

Several types of predisposition, some manifest as preferences for biological stimuli such as faces or biological motion, have been identified. Chicks and human infants have been studied in particular detail (for reviews see e.g. refs^2,31,32^). Such predispositions may depend on previous experience, but not necessarily experience of the object ultimately preferred. For example, handling and placement of chicks in running wheels in darkness for two hours is sufficient to trigger a predisposition to prefer a model of an adult fowl, even though the chicks have not previously seen it^33^. This is in contrast to the effect of training with a particular visual stimulus, as in the present experiments, which produces a specific preference for the stimulus through visual imprinting. The present results are consistent with the interpretation that low expression of gga-miR-130b-3p in the IMM is associated with a predisposition to learn well during subsequent visual imprinting training. One cannot say whether such a predisposition is related to any other predisposition because the requisite behavioural test was not performed. Mayer *et al*.^34^ have found that chicks with a predisposition to prefer an adult fowl displayed differential (lower) neuronal Fos-immunoreactivity in the IMM. Although lesion studies have shown that the IMM is not necessary for the adult fowl predisposition^35^, it is nevertheless of interest, and perhaps not surprising, to find further evidence of a functional link between a predisposition and the IMM.

Cytoplasmic CPEB-3 level was not significantly correlated with preference score. CPEB proteins exhibit bidirectional, microtubule-dependent movements, and it has been suggested that they might shuttle between soma and dendrites to repetitively capture and deliver mRNA transcript and modulate translation in particular parts of the neuron^36^. The fact that a learning-related change was found in M-CPEB-3 (mitochondrial-membrane fraction) but not C-CPEB-3 (cytoplasmic fraction) may indicate that learning-related up-regulation of dendritic mRNA delivery and local translation by CPEB-3 occurs preferentially in dendrites if M-CPEB-3 is enriched in dendritic components (see also below).

No significant changes were found for CPEB-1. As noted above, both CPEB-1 and CPEB-3 are targets of gga-miR-130b-3p action. However, one cannot yet confidently exclude the possibility of learning-related modulation of CPEB-1 in the IMM because it is subject to many influences and may exert an influence at other times after training. Co-regulated miRNAs can act conjointly^37,38^, and this may be the case for CPEB-1 and CPEB-3. They are also targets of other miRNAs, such as gga-miR-130c-3p (see http://www.mirdb.org/), which according to our profiling data may be up-regulated with learning (see Table 1). In addition, they may be subject to regulation by circular RNAs (see e.g. ref.^39^).

Little is known about the role of gga-miR-130b-3p in the brain. However, low levels of plasma miR-130b-3p are associated with vascular dementia in humans^40^, whilst its level is increased in the plasma of patients suffering from Huntington’s disease^41^. As regards other tissues, inhibition of miR-130b-3p appears to improve mitochondrial biogenesis signaling^42^ and overexpression of this miRNA reduces intracellular levels of ATP^43^. In agreement with these observations, we have shown learning-related increased mitochondrial dynamics and increased amounts of key mitochondrial proteins 24 h after training in the IMM^23,24,44^.

The CPEB family of proteins comprises four members (CPEB-1, CPEB-2, CPEB-3 and CPEB-4)^45^. CPEB-3 is the only family member to contain a prion-like domain^46^, a structure that can act as an epigenetic regulator or scaffold for subcellular organization^47^. CPEB-3 exists in two forms, soluble and aggregated. Soluble CPEB-3 is covalently attached to small ubiquitin-like modifier (SUMO) and functions as a repressor of translation. Removal of SUMO converts CPEB-3 to the aggregated form, which is amyloid-like, can promote translation, and has been proposed to be a functional prion regulating local synaptic protein synthesis necessary for maintenance of hippocampal long-term synaptic plasticity^48^. We have shown that another protein containing a prion-like domain is up-regulated in a learning-related way in the left IMM 24 h after imprinting training^23^. This protein is heterogeneous nuclear ribonucleoprotein A2/B1, which is involved in the delivery and translation of mRNAs into dendrites. Prion-like domain-containing proteins and dendritic translation processes therefore appear to have an important role in the memory underlying visual imprinting.

Much evidence indicates that the left and right IMM are involved in imprinting but take diverging physiological and molecular trajectories after training (see refs^5,14,15^). Lesion studies indicate that the right IMM but not the left is necessary for the formation of an additional memory store that is established outside the IMM several hours after the end of training^49–51^, and neuronal responsiveness to visual and auditory components of an imprinting stimulus evolves differently after training in the left and right sides of the IMM^52^. The subtle differences in Fig. 1A,B also hint at a hemispheric asymmetry in the role in imprinting of gga-miR-130b-3p.

In conclusion, we report that reduced gga-miR-130b-3p expression in a memory region may predict the readiness with which an animal learns in a given training procedure. In addition, the results indicate that a target of this miRNA, CPEB-3, in the membrane-mitochondrial fraction, is specifically related to the amount of learning that actually occurs as a result of training. This latter effect is lateralised, being restricted to the left IMM.

## Methods

### Training and testing

Fertile eggs (Cobb 500) were obtained from local poultry farm in Tbilisi, Georgia. Twenty-four batches of chicks were hatched and reared in darkness and trained with an imprinting stimulus for one hour as described previously^44^. In each batch there were <= 3 trained chicks and a control chick from the same hatch. At 22–28 h post-hatch, each chick to be trained was exposed in a running wheel to a training stimulus - a rotating red box^53^. The maternal call of a hen was played during this training procedure. Wheel turns caused by the chick measured training approach. Chicks received a preference test in running wheels 10 min after training by being shown sequentially the training stimulus and an alternative stimulus (a rotating blue cylinder); the maternal call was not played during the preference test.

The preference score was calculated as follows:

$${\rm{Preference}}\,{\rm{score}}=\frac{100\times ({\rm{approach}}\,{\rm{to}}\,{\rm{training}}\,{\rm{stimulus}})}{{\rm{approach}}\,{\rm{to}}\,{\rm{training}}\,{\rm{stimulus}}+{\rm{approach}}\,{\rm{to}}\,{\rm{alternative}}\,{\rm{stimulus}}}$$

A score of ~100 indicates strong learning, ~50 indicates poor learning. There are individual differences in chicks’ preference scores after a fixed period of training. This variation was used to run correlations between preference score on the one hand and expression of gga-miR-130b-3p, CPEB-1 or CPEB-3 on the other. The trained chicks achieved a range of preference scores: where possible, in each batch one chick was selected with a preference score >40 and <= 60 (poor learner); one with a preference score >60 and <= 80 (intermediate learner) and one with preference score >80 (good learner). In addition, there was one untrained chick in each batch.

Chicks were decapitated 24 h after training. Four pieces of tissue were removed, from the left and right IMM and from the left and right PPN, and immediately covered in dry ice. The locations of the IMM and PPN are shown in Solomonia *et al*.^22^, Fig. 1A,B; details of tissue removal from the IMM and PPN are described by Horn^54^ and Solomonia *et al*.^17^ respectively. All further procedures were conducted blind. All experimental procedures on live animals were approved by the Bioethics Committee of the I. Beritashvili Centre of Experimental Biomedicine and were conducted in compliance with the guidelines of that committee. Animal numbers were estimated from experience to be the minimum for adequate statistical analysis.

### RNA isolation

For profiling of miRNA, RNA was isolated from the left IMM of the chicks using an miRNeasy Mini Kit (Qiagen, 217004). For the quantitative measurement of miRNAs by RT-PCR the RNA was isolated by the same kit from all four brain regions studied. The concentration of RNA was measured by absorbance at wavelength 280/260 nm on Nanodrop.

### *μ*Paraflo™ MicroRNA microarray Assay

miRNA profiling experiments were done on the left IMMs of 5 good learner and 5 poor learner chicks using a service provider (LC Sciences). Profiling started with 1 µg of total RNA. Samples were 3′-extended with a poly(A) tail using poly(A) polymerase. An oligonucleotide tag was ligated to the poly(A) tail for later fluorescent dye staining. Hybridization was performed overnight on a *µ*Paraflo microfluidic chip using a micro-circulation pump (Atactic Technologies)^55,56^. Each detection probe on the microfluidic chip consisted of a chemically modified nucleotide coding segment, complementary to 998 *gallus gallus* miRNAs (from miRBase version 21, http://mirbase.org) in triplicates or other RNA (control) and a spacer segment of polyethylene glycol, to extend the coding segment away from the substrate. Detection probes were made by *in situ* synthesis using photogenerated reagent chemistry. The hybridization melting temperature was balanced by chemical modifications of the detection probes. Hybridization used 100 μL 6xSSPE buffer (0.9 M NaCl, 60 mM Na_2_HPO_4_, 6 mM EDTA, pH 6.8) containing 25% formamide at 34 °C. After RNA hybridization, tag-conjugating Cy3 dye was circulated through the microfluidic chip for dye staining. Fluorescence images were collected using a laser scanner (GenePix 4000B, Molecular Device) and digitized using Array-Pro image analysis software (Media Cybernetics). Data were analyzed by first subtracting the background and then normalizing the signals using a LOWESS (locally-weighted regression) filter^57^.

### miRNA measurement

The RNA fractions from IMM and PPN were reverse-transcribed by a Taqman MicroRNA Reverse Transcription Kit (Thermo Fisher Scientific 4366596). The amount of gga-miR-130b-3p was measured by a TaqMan™ assay kit from Thermo Fisher (Assay ID mmu481434_mir) using a Step One Real-Time PCR System (Applied Biosystems) and normalized to the amount of the two following miRNAs: gga-miR-221-3p and gga-miR-99a-5p (for selection of housekeeper microRNAs see Results). The amounts of housekeeper miRNAs were also measured using a TaqMan™ assay kit from Thermo Fisher (for gga-miR-221-3p, assay ID mmu481005_mir and for gga-miR-99a-5p, assay ID mmu478519_mir) The comparative CT (ΔΔCT) method was used to determine the relative target quantity in samples^58^. The same fraction of RNA, isolated from IMMs of untrained chicks, was used in all experiments as a reference sample. Each reaction was conducted in triplicate.

### Tissue fractionation for electrophoresis and immunoblotting

Samples were rapidly homogenized in 20 mM Tris-HCl (pH 7.4), 0.32 M sucrose, 1 mM ethylenediaminetetraacetic acid, 1 mM sodium orthovanadate, 10 mM sodium pyrophosphate, 0.5 mM methyleneglycol-bis(2-aminoethylether)-N,N,N′,N′-tetraaceticacid, and a cocktail of protease inhibitors (Sigma, P8340), and centrifuged at 1000 g for 10 min. The supernatant was further centrifuged at 15,000 g for 20 min. The resulting supernatant is referred to as the cytoplasmic fraction. The pellet was washed once and is referred to as the P2 mitochondrial-membrane fraction. A concentrated solution of sodium dodecylsulphate (SDS) was added to the cytoplasmic fractions to give a final concentration of 5%. The P2 mitochondrial-membrane fraction was also dissolved in 5% SDS. Protein concentrations were determined in quadruplicate using a micro bicinchoninic acid protein assay kit (Pierce). Amounts of CPEB-1 and CPEB-3 were measured in the cytoplasmic fraction (referred to as C-CPEB-1 and C-CPEB-3) and in the P2 mitochondrial-membrane fractions (M-CPEB-1 and M-CPEB-3).

Aliquots containing 30 μg of protein in 30 μl were subjected to SDS gel electrophoresis and western blotting as described previously^19^. After protein had been transferred onto nitrocellulose membranes, the membranes were stained with Ponceau S solution and analysed with Image J software (https://imagej.net/ImageJ) to confirm transfer and uniform gel loading. After Ponceau S staining the nitrocellulose membranes were cut in the range of desired molecular weight according to protein standards on the same gel and stained with the following well-characterized, commercially available primary antibodies: anti-CPEB-1 (ab 3465, Abcam) and anti-CPEB-3 (ab10883 Abcam) (datasheets available from https://www.abcam.com). Standard immunochemical procedures were performed using peroxidase-labelled secondary antibodies and Super Signal West Pico Chemiluminescent substrate (Pierce). Blots were exposed with intensifying screens to X-ray films pre-flashed with Sensitize (Amersham). Optical density of protein bands was measured using LabWorks 4.0 (UVP) software. The autoradiographs were calibrated using standard amounts of protein obtained either from the cytoplasmic or P2 mitochondrial-membrane fractions of the IMMs of a group of untrained chicks. Four standards (15, 30, 45 and 60 µg total protein) were applied to each gel. For these standards, the optical densities of bands immunostained for the corresponding protein (e.g. M-CPEB-3) were plotted against amount of protein; in all these standards, least squares regression showed a significant fit to a straight line and correlation coefficients ranged between 0.93 and 1.00. Optical density of each sample band was divided by optical density of the band for 30 μg of protein standard to give “relative amount of protein”^18^. Sample blots are shown in Fig. 3.

Data from experimental stained bands were not normalized with respect to actin or any other housekeeping protein because it cannot be guaranteed that such proteins are unaffected by imprinting^59–62^ for discussion of the unreliability of normalization to housekeeping proteins. In addition, study of housekeeping gene expression patterns in the IMM has also indicated that actin, GAPDH and other proteins are not reliable controls and that the left and right sides differ in expression stability^63^. Instead, we controlled loading by Ponceau S staining and calibration with protein standards and standardization using mean amount of protein in each batch (see below).

### Statistical analysis

Data from the IMM and PPN were analysed separately because the IMM is essential for imprinting and the PPN is not^14^; the latter region was therefore treated as a control as in previous studies^15^. A mixed regression model was fitted to the biochemical data, with fixed terms preference score and Side, and random terms Chick nested within Batch. Analysis was conducted using the lme function^64^ in R^65^. In addition to this main analysis, the left and right sides of the IMM and PPN were analysed separately to investigate laterality. Data shown in the figures have been standardized by discarding the batch-to-batch variation identified by fitting the Batch term and then adding back the overall mean to the residuals. After the profiling experiment, which was used as a screening procedure, probabilities <0.05 were taken as significant. Statistical tests are two-tailed unless stated otherwise. The R script used for statistical analysis is given in Supplementary Information.

## Electronic supplementary material

Table S1 and analysis software

Supplementary Tables S2-S4
