# Supplementary material for: Micro-RNAs, their target proteins, predispositions and the memory of filial imprinting
Source: Sci Rep. 2018 Nov 28;8:17444. doi: 10.1038/s41598-018-35097-w (PMC6262022; doi:10.1038/s41598-018-35097-w)
Supplement: Supplementary file 2 — Supplementary Tables S2-S4 [file 41598_2018_35097_MOESM2_ESM.pdf]

Micro-RNAs, their target proteins, predispositions and the memory of filial imprinting

Giorgi Margvelani, Maia Meparishvili, Tamar Kiguradze, Brian J McCabe, Revaz Solomonia

Supplementary Information
Supplementary Tables S2-S4

Supplementary Table S2. Standardized relative amount of gga-miR-130b-3p in PPN. Conventions as for Table 2.

Brain region	PPN, left and right combined		Left PPN		Right PPN	
Housekeeper	miR-221-3p	miR-99a-5p	miR-221-3p	miR-99a-5p	miR-221-3p	miR-99a-5p
**Untrained chicks**						
Mean	1.07	0.78	1.08	0.84	0.99	0.70
s.e. mean	0.07	0.02	0.08	0.03	0.08	0.02
df	8	8	9	8	9	10
**Trained chicks**						
Correlation, mi-RNA amount vs preference score	0.02	-0.12	-0.34	0.07	0.26	-0.38
df	9	9	10	10	9	9
P	0.95	0.72	0.28	0.83	0.44	0.25
y-intercept at preference score 100	1.06	0.81	1.02	0.85	1.11	0.75
s.e. y-intercept	0.08	0.06	0.09	0.07	0.11	0.07
Comparison, y-intercept at preference score 100 vs mean for untrained chicks						
t	-0.09	0.42	-0.51	0.11	0.87	0.70
df	16.7	10.3	18.8	13.2	16.8	11.0
P	0.93	0.69	0.62	0.91	0.40	0.50
y-intercept at preference score 50	1.05	0.83	1.12	0.83	0.98	0.85
SE y-intercept	0.08	0.06	0.09	0.07	0.10	0.06
Comparison, y-intercept at preference score 50 vs mean for untrained chicks						
t	-0.15	0.94	0.31	-0.13	-0.08	2.31
df	15.2	10.4	17.0	12.9	15.3	11.0
P	0.88	0.37	0.76	0.89	0.94	<u>0.04</u>
**Residual regression variance/variance (untrained)**	0.47	6.39	0.31	2.64	1.00	3.09
P	0.28	<u>0.016</u>	0.084	0.18	1.00	0.10

Supplementary Table S3. Standardized relative amount of CPEB-1 in PPN. Conventions as for Table 3.

Brain region	PPN, left and right combined		Left PPN		Right PPN	
Protein	M-CPEB-1	C-CPEB-1	M-CPEB-1	C-CPEB-1	M-CPEB-1	C-CPEB-1
**Untrained chicks**						
Mean	1.10	1.19	1.10	1.17	1.09	1.09
s.e. mean	0.08	0.15	0.07	0.15	0.11	0.19
df	7	6	7	7	7	6
**Trained chicks**						
Correlation, protein amount vs preference score	0.15	0.18	0.39	0.20	-0.17	0.07
df	11	12	12	12	11	12
P	0.61	0.53	0.16	0.50	0.57	0.81
y-intercept at preference score 100	1.03	1.30	1.10	1.46	0.95	1.14
s.e. y-intercept	0.07	0.18	0.09	0.27	0.09	0.16
Comparison, y-intercept at preference score 100 vs mean for untrained chicks						
t	-0.62	0.51	-0.05	0.97	-1.01	0.22
df	15.87	17.30	18.93	17.85	15.36	13.62
P	0.54	0.62	0.96	0.35	0.33	0.83
y-intercept at preference score 50	0.97	1.10	0.86	1.12	1.05	1.07
s.e. y-intercept	0.08	0.20	0.10	0.31	0.11	0.18
Comparison, y-intercept at preference score 50 vs mean for untrained chicks						
t	-1.11	-0.35	-1.88	-0.14	-0.27	-0.07
df	17.64	17.59	17.93	16.06	17.71	17.99
P	0.28	0.73	0.08	0.89	0.79	0.95
Residual regression variance/variance (untrained)	0.47	1.30	1.10	2.61	0.57	0.65
P	0.24	0.78	0.94	0.20	0.40	0.50

Supplementary Table S4. Standardised relative amount of CPEB-3 in PPN. Conventions as for Table 4

Brain region	PPN, left and right combined		Left PPN		Right PPN	
Protein	M-CPEB-3	C-CPEB-3	M-CPEB-3	C-CPEB-3	M-CPEB-3	C-CPEB-3
**Untrained chicks**						
Mean	1.12	1.09	1.16	1.08	1.08	1.00
s.e. mean	0.04	0.10	0.08	0.13	0.09	0.15
df	6	6	7	7	6	6
**Trained chicks**						
Correlation, protein amount vs preference score	-0.41	0.10	-0.19	0.21	-0.52	-0.07
df	12	12	12	12	12	12
P	0.14	0.73	0.52	0.46	0.06	0.81
y-intercept at preference score 100	1.00	1.17	1.06	1.25	0.96	1.10
s.e. y-intercept	0.13	0.12	0.13	0.16	0.14	0.12
Comparison, y-intercept at preference score 100 vs mean for untrained chicks						
t	-0.86	0.55	-0.62	0.85	-0.73	0.53
df	14.4	17.6	18.0	18.9	17.9	13.7
P	0.40	0.59	0.54	0.41	0.47	0.61
y-intercept at preference score 50	1.25	1.09	1.20	1.03	1.29	1.16
s.e. y-intercept	0.14	0.14	0.15	0.19	0.15	0.14
Comparison, y-intercept at preference score 50 vs mean for untrained chicks						
t	0.88	0.04	0.26	-0.22	1.12	0.76
df	13.9	17.5	16.4	17.9	17.0	18.0
P	0.39	0.97	0.80	0.83	0.28	0.46
Residual regression variance/variance (untrained)	3.33	1.43	1.57	1.28	0.63	0.60
P	0.14	0.68	0.56	0.76	0.46	0.42
